# Impaired Anorectal Afferents Is a Potential Pathophysiological Factor Associated to Functional Anorectal Pain

**DOI:** 10.3389/fneur.2020.577025

**Published:** 2020-10-09

**Authors:** Qi Zhang, Yanni Liu, Qiong Zhang, Yuqing Zhang, Sangsang Wu, Bin Jiang, Min Ni

**Affiliations:** ^1^Graduate School, Nanjing University of Chinese Medicine, Nanjing, China; ^2^Baoji City Hospital of Traditional Chinese Medicine, Baoji, China; ^3^Shuyang County Hospital of Traditional Chinese Medicine, Suqian, China; ^4^National Centre of Colorectal Disease, Nanjing Hospital of Chinese Medicine, Nanjing, China

**Keywords:** functional anorectal pain, evoked potential, afferent pathway, pathophysiology, anorectal manometry

## Abstract

**Background/Aims:** Functional anorectal pain (FARP) is a functional gastrointestinal disease, which belongs to chronic pelvic floor pain. The mechanisms of its development are not fully understood. We designed this experiment to evaluate the characteristics of rectal sensory evoked potential (RSEP) and anorectal manometry (ARM) in this population, so as to explore the pathophysiology of FARP.

**Methods:** The rectal sensory evoked potentials (RSEP) and anorectal manometry (ARM) were performed in 23 patients with FARP and 23 healthy controls. The correlation between the two measurements was investigated.

**Results:** The results of RSEP showed that (1) the median latency to the first positive peak was 69.2 ± 15.9 ms in patients, compared with 46.5 ± 5.8 ms in controls (*P* = 0.000). (2) The amplitude of evoked potential peaks in the FARP patients was significantly lower than the healthy controls (P1/N1: *P* = 0.049; N1/P2: *P* = 0.010). (3) Compared with the controls, the patients showed a lower maximum voluntary squeeze pressure (*P* = 0.009), lower rectum (*P* = 0.007), and anal sphincter pressures (*P* = 0.000) during strain; and increased maximum tolerance threshold to rectal distention (*P* = 0.000). (4) The resting pressure of the anal sphincter was correlated with the peak amplitude of the RSEP (P1/N1: *r* = 0.537, *P* = 0.039; N1/P2: *r* = 0.520, *P* = 0.047). Considering the different pathophysiological mechanisms of levator ani syndrome and proctalgia fugax, we analyzed data on unspecified functional anorectal pain and obtained similar results.

**Conclusions:** The RSEP can be used to evaluate the state of afferent pathways in FARP patients; The longer latency and lower peak amplitude of RSEP indicate the functional defects of the anorectal afferent pathway. It can provide an objective evidence for the neuropathy of FARP. In addition, the pathophysiology of FARP is also associated with pelvic floor muscle motor and coordination dysfunction. The correlation between the peak amplitude of the RSEP and the resting pressure of the anal sphincter suggests that there seems to be a correlation between anal pressure and the afferent signaling pathway in patients with FARP.

## Introduction

Functional anorectal pain (FARP) is a disabling disease and can be caused by a variety of factors. Rome IV divides FARP into three subtypes: proctalgia fugax, levator ani syndrome, and unspecified functional anorectal pain (1–3). Levator ani syndrome and unspecified functional anorectal pain are distinguished by whether there is pain when pulling the levator ani muscle backward (4). A previous survey of householders in the United States found that the prevalences of the anorectal pain, levator ani syndrome, and proctalgia fugax were 11.6, 6.6, and 8%, respectively (5), and most of them were women. The patients are often accompanied with mental and emotional disorders that seriously affect the quality of life and mental health of patients.

The pathophysiology of FARP is still unclear. At present, it is generally believed that the over contraction and high tension of pelvic floor muscle are one of the important mechanisms. Levator ani syndrome is considered to be closely related to pelvic floor spasm and can usually be relieved by the biofeedback therapy (6, 7). Rao SS et al. reported that paroxysmal anal hyperkinesis was an outstanding characteristic feature of proctalgia fugax (8). However, in our clinical practice, we have observed that functional anorectal pain is also present in patients with hypotensive pelvic floor muscle, and this group of patients usually have sensation of downward bloating in their anus, and most of them are women.

Abnormal regulation of the nervous system may also lead to FARP. The function of gastrointestinal tract is controlled by the central nervous system, autonomic nervous system, and enteric nervous system. There is a biphasic regulatory pathway between the digestive tract and the central nervous system (9); the brain-gut dysfunction has been proven to be closely related to gastrointestinal disorders (3, 10, 11). The visceral sensory function is one of the important contents in the brain-gut axis research, and heightened visceral perception or visceral hypersensitivity has long been considered as a potential pathogenesis of functional bowel disease (12, 13), especially IBS (14, 15). The mechanism of visceral hypersensitivity has not been fully elucidated. It may be result from the sensitization of nerve afferent pathways originating from the gastrointestinal tract (16). At present, there is a lack of reports in the literature on the sensory abnormality in patients with functional anorectal pain.

Neurophysiological tests can provide useful information regarding the integrity of neuronal innervation and neuromuscular function of the gut. During the last few years, some techniques have provide information regarding gut-brain communication such as Positron Emission Tomography (PET) or functional magnetic resonance imaging (fMRI). Recently, RSEP has been introduced as a new technique that can provide an quantifiable method to evaluate the connections between the afferent tracts, spinal cord, and the cerebral cortex (9). In this study, our aim was to prospectively evaluate and compare RSEP following rectal electrical stimulation and ARM in FARP patients, and healthy controls. So as to evaluate the anorectal sensory and motor functions and neural afferent pathways in FARP patients, and explore possible mechanisms of FARP.

## Materials and Methods

### Subjects

This study was reviewed and approved by the Ethics Committee of Nanjing Hospital of Chinese Medicine (KY2018004). The participants provided their written informed consent to participate in this study. Twenty three patients with FARP were recruited from patients seen in the Anorectal Clinic of Nanjing Hospital of Chinese Medicine. All patients met the Rome IV criteria for functional anal pain. Patients with a history of anorectal surgery, secondary anorectal pain with clear etiology, serious gastrointestinal diseases or oral drugs affecting gastrointestinal functions, serious neurological diseases, spinal injury, or surgery were excluded, as were pregnant women.

In order to exclude the influence of age and gender, the control group consisted of 23 healthy people whose sex and age matched with the patients. These healthy people did not have FARP and diseases listed below: gastrointestinal diseases, severe perianal diseases, or severe cardiovascular. Individuals with any cerebrovascular, urological, gynecological, orthopedic, or malignant tumors were also excluded.

### Rectal Sensory Evoked Potentials After Electrical Stimulation

For rectal stimulation, a St. Mark's stimulation electrode was fixed at the tip on the index finger while the other electrode was located at the root of the index finger, and the electrodes were connected to an electrical stimulator (Oxford Instruments, Oxford, Britain). For recording of evoked potential, an active electrode was placed on the subject's central scalp (Cz); a reference electrode was placed on the forehead (Fz); and a grounding electrode was placed on the ankle. This was in accordance with the International Electroencephalogram Society standard (17). The test was conducted in a dark room, unnecessary electrical equipment were turned off to avoid electromagnetic interferences. Subjects were requested to lie relaxed with their eyes closed. The examiner put the index finger into the rectum of the patient with the stimulation electrode located at 9 o'clock of the lithotomy position and 5 cm from the anal margin. The sensory threshold was defined as the minimum stimulation intensity of the patient's perception, and the tolerance threshold was defined as the maximum tolerable intensity. The final stimulus intensity was set at 75% of the mean tolerance threshold. The evaluation was repeated three times to ensure consistency. The electric stimuli were composed of 100 pulses with a pulse width of 0.2 ms, frequency of 1 Hz. The impedance between the electrodes was maintained below 5 kΩ. The RSEP data were obtained using the Oxford myoelectricity-evoked potential apparatus (Oxford Instruments, Oxford, Britain). The sensitivity of the amplifier was 100 μv/div and the recording bandwidth was set to 1~500 Hz.

**Figure 1:**
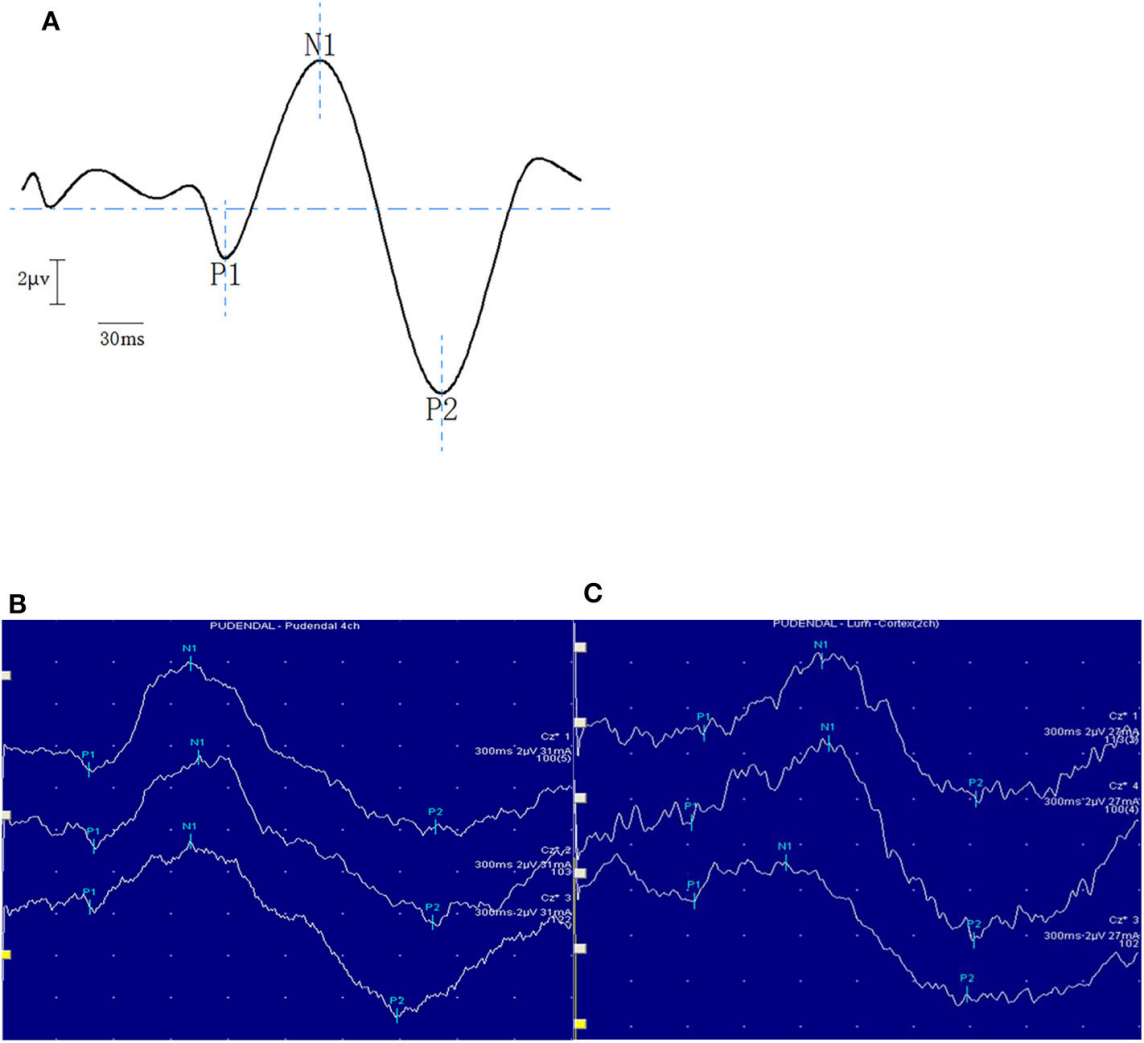
The panel above is the schematic diagram of typical waveforms of RSEP **(A)**. The bottom panel shows typical examples of RSEP responses in a healthy subject **(B)** and in a patient with FARP **(C)**.

After electrical stimulation, three typical main waveforms could be recorded (18), which are labeled P1, N1, and P2, respectively (Figure 1A). Positive waves with downward amplitude were represented by P, and negative waves with upward amplitude were represented by N. The time from the start of the stimulus to the appearance of the peak was called the latency. The voltage difference between two consecutive peaks was referred as the amplitude, represented by P1/N1 and N1/P2.

### Anorectal Manometric Testing

Anorectal manometry (ARM) was performed via an 8-channel radially distributed water perfusion catheter with a latex balloon attached at the tip of the catheter (Medtronic Synectics, Sweden). Before anorectal manometry, all participants were required to empty their stools to ensure that the rectum was empty. The patient lay on the left side and remained relaxed; the examination was performed by a well-trained doctor. Following measurements were made: rectal motility functions (anal resting pressure, maximum voluntary squeeze pressure, anal sphincter pressure during straining, and rectal pressure during straining) and rectal sensory functions (first sensation, urge to defecate, and maximum tolerance volume of rectal balloon distention). Other measurements included the length of functional anal canal, rectum anal inhibitory reflex (RAIR), contractile reflex of the anorectum during coughing (cough reflex) and defecation reflex. The procedures and measurements followed the standards of previous studies (19, 20).

### Statistical Analyses

SPSS 20.0 version was used for statistical analyses. The data are expressed as mean ± standard deviation with 95% confidence interval. Counting data is expressed by frequencies and percentages, Fisher's exact tests is used for comparison between groups. The Mann Whitney *U*-test was used to compare the latency and amplitude of RSEP waves, and parameters of anorectal manometry between the FARP patients and healthy controls. The correlations between the parameters of RSEP and the parameters of anorectal manometry were analyzed by Pearson or Spearman correlation analysis.

## Results

### Demographics

**Figure 2:**
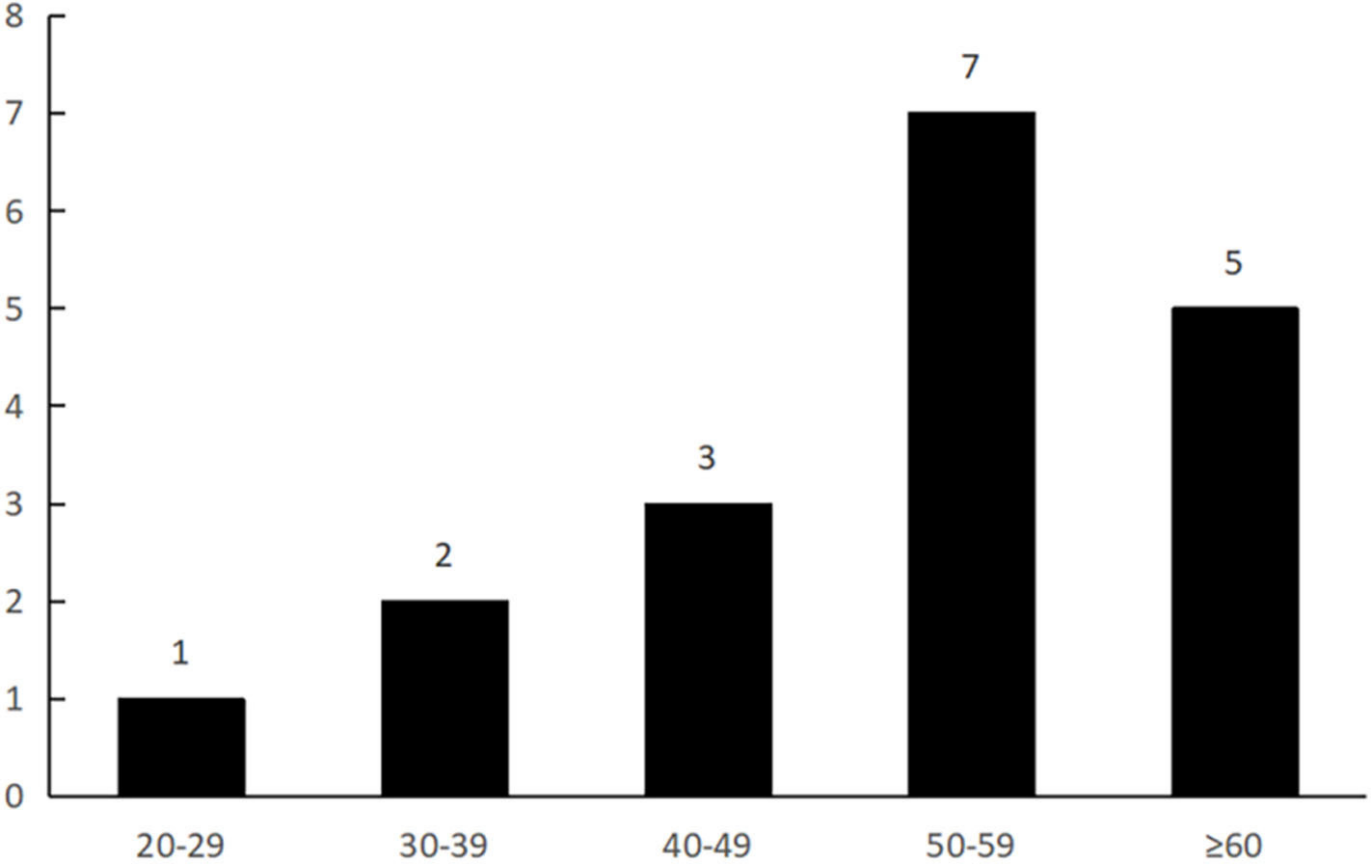
This figure shows that most of the female FARP patients were over 40 years old, and mainly distributed in the age group of 50–59 years old.

All 23 patients with FARP were enrolled in the study. There was 21/23 patients with unspecified functional anorectal pain (Unspecified-FARP), and 1/23 patient with proctalgia fugax (PF),1/23 patient with levator ani syndrome(LAS) The average age of the patients was 52.48 (±12.98) years old and gender distribution of the two groups was 18/5 (F/M). There was no significant difference in height, weight or BMI between the patients and healthy controls (Table 1). The female patients with FARP were divided into five groups according to their ages: group I (20–29 years old), group II (30–39 years old), group III (40–49 years old), group IV (50–59 years old), and group V (60 years old and above) (Figure 2). Most of the female FARP patients were over 40 years old, mainly distributed in the age group of 50–59 years old. Among 21 patients with unspecified functional anorectal pain, 2 female FARP patients had a history of multiple vaginal deliveries.

**Table 1:** Demographics of FARP patients and healthy controls.

	**FARP (*n =* 23)**	**Controls (*n =* 23)**	***p***
Gender (female)	18	18	–
Age	52.48 ± 12.98	49.91 ± 12.07	0.422
Height (m)	1.63 ± 0.08	1.66 ± 0.70	0.205
Weight (kg)	62.09 ± 9.38	61.65 ± 6.83	0.965
BMI (kg/m^2^)	23.46 ± 3.18	22.36 ± 1.62	0.287

*Values are given as mean (±SD)*.

### Rectal Sensory Evoked Potential

A reproducible RSEP pattern was recorded in all subjects after rectal stimulation. Typical examples of the morphology of RSEP response are shown in Figures 1B,C. The latencies and amplitudes of each component of the RSEP of control group are shown in Table 2, and there were no differences among RSEP components between men and women subjects.

**Table 2:** Latencies and amplitude of each component of the RSEP response following Rectal Stimulation in healthy controls.

	**Total (*n =* 23)**	**Males (*n =* 5)**	**Female (*n =* 18)**	***p***
P1 latency (ms)	46.50 ± 5.77	46.18 ± 2.77	46.59 ± 6.42	0.914
N1 latency (ms)	101.32 ± 17.78	102.74 ± 23.69	100.93 ± 16.62	0.801
P2 latency (ms)	193.70 ± 34.42	203.98 ± 28.02	190.85 ± 36.18	0.446
P1/N1 amplitude (uV)	4.67 ± 2.35	5.44 ± 1.99	4.46 ± 2.45	0.538
N1/P2 amplitude (uV)	6.57 ± 2.78	7.50 ± 2.76	6.31 ± 2.80	0.491

*Values are given as mean (±SD)*.

The latency of P1 was significantly longer in the FARP patients than the healthy controls (69.20 ± 15.91 vs. 46.50 ± 5.77, *P* < 0.01). There was no significant difference in N1 and P2 latency between the two groups. The amplitude of each wave in the FARP patients was significantly lower than the healthy controls (P1/N1: 3.66 ± 2.10 vs. 4.67 ± 2.35; N1/P2: 4.25 ± 3.07 vs. 6.57 ± 2.78, *P* < 0.01). In order to further explore the mechanism of unspecified functional anorectal pain, we compared the RSEP examination of unspecified-FARP patients with that of healthy controls (Table 3).

**Table 3:** Latencies and amplitude of each component of the RSEP response following Rectal Stimulation in patients.

	**FARP (*n =* 23)**	**Unspecified-FARP (*n =* 21)**	**Controls (*n =* 23)**	***p***	
				**FARP**	**Unspecified-FARP**
P1 latency (ms)	69.20 ± 15.91	68.53 ± 16.13	46.50 ± 5.77	<0.001	<0.001
N1 latency (ms)	112.28 ± 29.35	113.73 ± 30.06	101.32 ± 17.78	0.199	0.155
P2 latency (ms)	168.64 ± 44.57	172.67 ± 44.58	193.70 ± 34.42	0.056	0.133
P1/N1 amplitude (uV)	3.66 ± 2.10	3.47 ± 1.75	4.67 ± 2.35	0.049	0.030
N1/P2 amplitude (uV)	4.25 ± 3.07	4.29 ± 3.08	6.57 ± 2.78	0.010	0.010

*Values are given as mean (±SD)*.

### Anorectal Manometry

All 15 of the 23 FARP patients performed anorectal manometry. After comparing the components of ARM between the FARP patients and the healthy controls, we found that the maximum voluntary squeeze pressure in the FARP patients was lower than the healthy controls (107.37 ± 32.53 vs. 135.61 ± 19.13, *P* < 0.01). Meanwhile, the FARP patients showed a significantly lower anal sphincter pressure and rectal pressure during straining (*P* < 0.01). There was no significant difference between the two groups in resting anal pressure, sphincter length. In addition, analrectal manometry results in 13 patients with unspecified-FARP were analyzed, and similar results were obtained (Table 4). All the data of 15 FARP patients who completed the two examinations are shown in Table 5. Among the 15 patients, patients with levator ani syndrome and proctalgia fugax have significantly higher anal resting pressure (93 and 80 mmHg, respectively).

**Table 4:** Anorectal manometric profiles in patients and healthy controls.

	**FARP (*n =* 15)**	**Unspecified-FARP (*n =* 13)**	**Controls (*n =* 23)**	***p***	
				**FARP**	**Unspecified-FARP**
Resting pressure	57.93 ± 16.38	53.54 ± 12.21	59.00 ± 6.52	0.836	0.328
Squeeze pressure	107.37 ± 32.53	103.23 ± 32.56	135.61 ± 19.13	0.009	0.004
Sphincter length	3.23 ± 0.20	3.08 ± 0.28	3.29 ± 0.13	0.145	0.974
Anal sphincter pressureduring straining	30.33 ± 13.67	29.69 ± 14.53	42.13 ± 7.85	0.007	0.012
First sensation	28.67 ± 16.42	29.23 ± 17.54	22.17 ± 7.36	0.442	0.494
Urge	71.33 ± 25.32	71.54 ± 26.09	74.35 ± 16.47	0.497	0.494
Maximum tolerable volume	121.33 ± 48.24	119.23 ± 51.71	185.22 ± 45.61	<0.001	<0.001
Rectal pressure during straining	30.87 ± 17.74	27.15 ± 14.76	55.43 ± 13.67	<0.001	<0.001

*Values are given as mean (± SD)*.

**Table 5:** The data of 15 FARP patients who completed the two examinations.

**Diagnosis**	**Gender**	**Age**	**P1 (ms)**	**N1 (ms)**	**P2 (ms)**	**P1/NI (uV)**	**N1/P2 (uV)**	**Resting pressure (mmHg)**	**Squeeze pressure (mmHg)**	**Sphincter length (cm)**	**Anal sphincter pressure during straining (mmHg)**	**Rectal pressure during straining (mmHg)**	**First sensation (ml)**	**Urge (ml)**	**Maximum tolerable volume (ml)**	**Defecation reflex**	**RAIR (ml)**
Unspecified-FARP	F	33	69.0	110.4	127.2	2.3	1.5	43	80	3	37	18	20	80	130	Abn.	10
	F	76	58.4	84.8	134.0	3.1	3.7	52	88	3.1	12	8	20	70	100	Abn.	10
	F	54	63.6	87.8	182.0	4.3	7.8	66	80	3.1	42	32	20	30	40	Norm.	10
	F	54	55.6	73.0	146.6	2.8	3.2	56	87	3.0	17	12	10	50	130	Abn.	10
	F	53	95.7	167.1	243.9	3.5	5.9	70	104	3.0	47	8	30	80	120	Norm.	20
	F	67	52.8	139.2	189.3	2.4	2.0	52	72	3.1	22	17	20	70	80	Norm.	20
	F	55	80.7	131.1	210.9	4.2	3.2	42	90	3.2	13	20	20	80	130	Abn.	20
	F	48	63.6	83.1	121.2	2.6	5.7	65	147	3.4	43	39	40	80	100	Norm.	30
	M	59	67.8	92.7	136.5	1.3	1.6	53	149	3.3	22	36	20	80	180	Abn.	20
	F	72	104.4	158.4	219.0	3.8	2.0	38	104	3.3	12	36	20	50	80	Norm.	20
	M	58	34.4	58.4	76.2	2.0	1.7	66	156	3.7	35	51	30	50	120	Norm.	10
	F	43	60.3	112.2	170.4	2.4	2.8	62	131	3.3	55	50	70	140	250	Norm.	10
	F	62	75.3	110.4	132.9	1.1	1.4	31	53	3.1	29	26	60	70	90	Norm.	10
LAS	M	55	65.0	83.4	131.0	9.3	6.8	93	148	3.4	30	40	20	50	130	Abn.	20
PF	F	52	87.6	110.7	121.8	2.0	0.96	80	121	3.4	39	70	30	90	140	Abn.	10

*LAS, levator ani syndrome; PF, proctalgia fugax; F, female; M, male; Norm., normal; Abn., abnormal*.

In terms of the rectal sensory function, rectal maximum tolerable volume was reduced in the FARP patients in comparison with the healthy controls (121.33 ± 48.24 vs. 185.22 ± 45.61, *P* < 0.01).There was no significant difference between the two groups in first sensation threshold or the urge to defecate threshold (Table 4).

RAIR was present in all patients and all controls. The mean volume of rectal distention needed to initiate RAIR in the healthy controls was 12.63 ± 5.03 mL, while patients needed larger volumes to elicit RAIR (15.33 ± 6.40; *P* < 0.05).

7/15 patients who received ARM showed abnormal defecation reflex, including the patient with levator ani syndrome. However, only one of the healthy controls showed abnormal defecation reflex. There is a significant difference between the two groups (*P* < 0.01). Cough reflex was normal in all subjects.

### The Correlation Between RSEP and Anorectal Manometry

**Figure 3:**
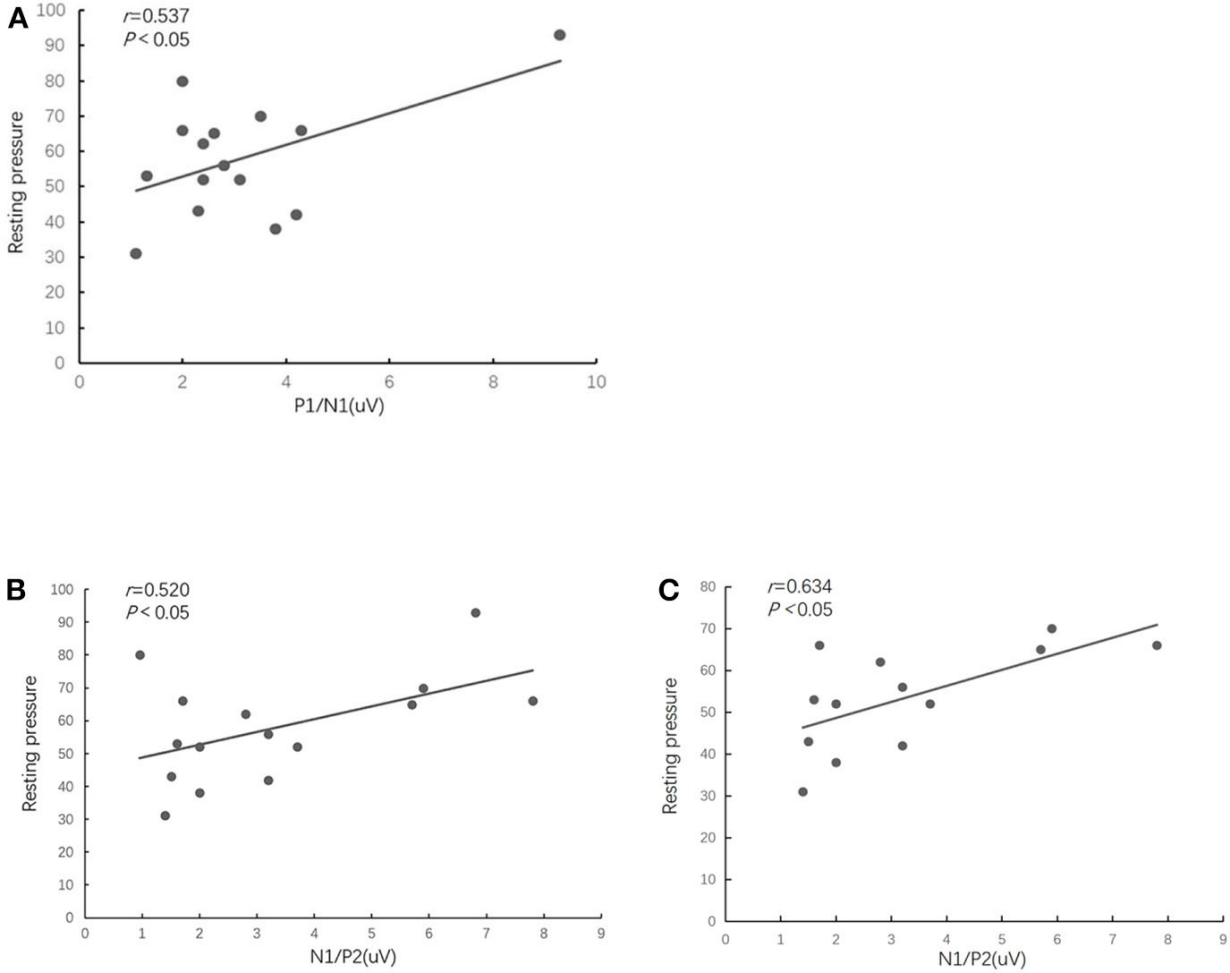
The scatterplot shows that the FARP patients' resting pressure of the anal sphincter was positively correlated with the amplitude of P1/N1 **(A)** and the amplitude of N1/P2 of RSEP **(B)**. There was a significant positive correlation between resting pressure and amplitude of N1/P2 in patients with unspecified-FARP **(C)**.

The Pearson's correlation analysis showed that resting pressure of the anal sphincter of FARP patients was positively correlated with the amplitude of P1/N1 (*r* = 0.537, *P* = 0.039) (Figure 3A)and the amplitude of N1/P2 (*r* = 0.520, *P* = 0.047) (Figure 3B). There was no significant correlation between any of RSEP parameters and rectal inhibitory reflex (RAIR) or any of rectal sensory parameters (the first sensation, urge, or maximum tolerance). In addition, we analyzed the relationship between RSEP and ARM parameters in patients with unspecified-FARP and found that there was a significant positive correlation between resting pressure and amplitude of N1/P2 (*r* = 0.634, *P* = 0.020) (Figure 3C). There was no significant correlation between the parameters of the two examinations in healthy control group.

## Discussion

Chronic anorectal pain is a refractory functional gastrointestinal disease. The Rome Classification provides well-defined diagnostic criteria for functional anorectal pain. However, it is still unclear of the pathophysiology of FARP and a huge challenge to treat FARP, though some advances in the treatment of functional anorectal pain have made recently (21–24). The pathophysiology of functional gastrointestinal diseases is closely related to the neuronal interactions between the brain and gut. RSEP provided objective data regarding the integrity of the afferent pathways. In this study, we found that latency of P1 of RSEP in the FARP patients were significantly longer and the amplitude of each waves were significantly decreased when compared to healthy controls.

ARM is widely used for the detection of abnormalities of sphincter function and rectoanal coordination (25). We found that the anorectal pressure during squeeze or strain, maximum tolerable volume to rectal distension were significantly reduced in 15 FARP patients in this study. After analyzing data of the 15 FARP patients, we found that different subtypes of FARP had different resting pressure characteristics, For example, 2/13 unspecified-FARP showed lower resting pressure while other unspecified-FARP FARP patients haven't change. But the PF and LAS patients showed higher anal resting pressure and abnormal anal relaxation reflex, which was consistent with the previous studies (8, 26), and pain in these patients might be related to the excessive activity of anal sphincter. These patients reported a sensation of downward bloating in their anus and most of them were middle-aged or older women. One possible mechanism might be attributed to pelvic floor muscle dysfunction. This might be closely related to vaginal delivery, age, and chronic constipation. Egorov et al. (27) reported that the pelvic muscle function decreased with age, and tissue elasticity decreased with multiple vaginal deliveries. In our study, 2 female FARP patients had a history of multiple vaginal deliveries. This might also be related to laceration of levator ani caused by natural labor in some women (28). Compared with the healthy controls, patients with FARP have a higher rate of abnormal defecation reflex, which seems to suggest that anorectal discordance may be one of the mechanisms leading to FARP.

Somatosensory EPs (SEPs) has advantages over other brain imaging techniques given its relative portability, inexpensiveness, availability. It can be used to evaluate the somatosensory pathway at the peripheral, spinal, cortical, and subcortical level (16). By stimulating the rectum and recording the potential changes of neurons in the cerebral cortex one can investigate the nerve transfer function from the rectum to the pelvic floor nerve and then to the spinal cord and the cerebral cortex. RSEP has been carried out in healthy people (29) and the fluctuation range of the data in the healthy control group in our study was basically the same as the above study. The RSEP has been widely used to evaluate the visceral sensitivity mechanism in IBS. Chan et al. found that compared to healthy subjects, irritable bowel syndrome patients demonstrated higher prevalence of cerebral evoked potential early peaks postprandially, and uniformly shorter cerebral evoked potential latencies both before and after feeding (30). Conversely, the prolongation of SEP latency has been reported in fecal incontinence and constipation (31, 32). This is thought to be related to receptor dysfunction, reduced activation of afferent nerves, or slowed peripheral nerve conduction (28). In our study, the FARP patients showed a longer latency of P1 in the RSEP. This finding once again demonstrated the impairment of the afferent pathway in FARP. The mechanism might be similar to fecal incontinence and constipation.

Conscious perception implies sensory connectivity between the rectum and the brain (33). Sensory threshold to rectal distention is also an important indicator of the rectal-brain afferent pathway. Törnblomd et al. compared the rectal sensory thresholds between IBS patients and healthy people, and found that the sensory thresholds in IBS patients were decreased (34). The increase of rectal threshold is a manifestation of rectal hyposensitivity, which is closely related to the disorder of the hindgut function, and usually manifested as fecal incontinence and constipation (35). The rectal sensory function has not been investigated in FARP. In our study, the FARP patients showed lower maximum tolerance threshold. Although the sensory threshold level in the ARM test can reflect the state of rectal sensory function to a certain extent, it has higher requirements on the cooperation degree and cognition of the test, and there may be some subjective deviations as well. In contrast, the RSEP is a more sensitive detection method that responds to the sensory pathways. The correlation between the resting pressure of the anal sphincter and the amplitude of the RSEP implied that there may be a relationship between anal sphincter pressure and neuropathy. Which still remains to be further studied.

The RAIR is a phenomenon in which the anal internal sphincter is temporarily relaxed due to the transient distension of the rectum. It plays an important role in bowel control and defecation (36). Studies have shown that the presence of RAIR is unrelated to the integrity of spinal cord and brain nerve centers, but requires a complete intramural pathway (37). Beuret-blanquart et al. have shown that the RAIR reflex appears to be controlled by autonomic neural pathways, especially the parasympathetic nervous system (38, 39). In our study, RAIR was present in 15 FARP patients after ARM, although FARP patients need larger volumes to elicit RAIR, which may indicate the integrity of intramural pathway in these patients. The difference in eliciting volume may be related to rectal compliance and sensory function abnormalities.

The limitations of our study include the small sample size and a comparative study among different subtypes could not be performed. Also, in this study, we didn't evaluat efferent pathways in FARP patients, and also deserves further study.

In conclusion, rectal sensory evoked potential is safe and well-tolerated and appears to be a useful technique for the detection of neuropathy, the test can provide an objective evidence for neuropathy and a new dimension toward our understanding of the mechanisms of FARP.

## Data Availability Statement

The raw data supporting the conclusions of this article will be made available by the authors, without undue reservation.

## Ethics Statement

The studies involving human participants were reviewed and approved by Ethics Committee of Nanjing Hospital of Chinese Medicine. The patients/participants provided their written informed consent to participate in this study.

## Author Contributions

MN and BJ conceived and designed research. QiZ and YL conducted research and wrote the initial paper. QioZ, YZ, and SW collected date. MN revised the paper and had primary responsibility for final content. All authors read and approved the final manuscript.

## Conflict of Interest

The authors declare that the research was conducted in the absence of any commercial or financial relationships that could be construed as a potential conflict of interest.

## References

[1] Rao SS Bharucha AE Chiarioni G Felt-Bersma R Knowles C Malcolm A Functional anorectal disorders. Gastroenterology. (2016) 150:1430–42. 10.1053/j.gastro.2016.02.009 PMC5035713 27144630

[2] Simren M Palsson OS Whitehead WE. Update on rome IV criteria for colorectal disorders: implications for clinical practice. Curr Gastroenterol Rep. (2017) 19:15. 10.1007/s11894-017-0554-0 28374308 PMC5378729

[3] Drossman DA Hasler WL. Rome IV—functional GI disorders: disorders of gut-brain interaction. Gastroenterology. (2016) 150:1257–61. 10.1053/j.gastro.2016.03.035 27147121

[4] Bharucha AE Lee TH Anorectal and pelvic pain. Mayo Clin Proc. (2016) 91:1471–86. 10.1016/j.mayocp.2016.08.011 27712641 PMC5123821

[5] Drossman DA Li Z Andruzzi E Temple RD Talley NJ Thompson WG. U.S. householder survey of functional gastrointestinal disorders. Prevalence, sociodemography, and health impact. Dig Dis Sci. (1993) 38:1569–80. 10.1007/BF01303162 8359066

[6] Chiarioni G Nardo A Vantini I Romito A Whitehead WE Biofeedback is superior to electrogalvanic stimulation and massage for treatment of levator ani syndrome. Gastroenterology. (2010) 138:1321–9. 10.1053/j.gastro.2009.12.040 20044997 PMC2847007

[7] Grimaud J-C Bouvier M Naudy B Guien C Salducci J. Manometric and radiologic investigations and biofeedback treatment of chronic idiopathic anal pain. Dis Colon Rectum. (1991) 34:690–5. 10.1007/BF02050352 1855425

[8] Lascano AM Lalive PH Hardmeier M Fuhr P Seeck M Clinical evoked potentials in neurology: a review of techniques and indications. Neurol Neurosurg Psychiatry. (2017) V88N8:688–96. 10.1136/jnnp-2016-314791 28235778

[9] Remes-Troche JM Tantiphlachiva K Attaluri A Valestin J Yamada T Hamdy S. A bi-directional assessment of the human brain-anorectal axis. Neurogastroenterol Motil. (2011) 23:240–8. 10.1111/j.1365-2982.2010.01619.x 20964791 PMC3035753

[10] Greenwood-Van Meerveld B Johnson AC Grundy D Gastrointestinal physiology and function. Handb Exp Pharmacol. (2017) 239:1–16. 10.1007/164_2016_118 28176047

[11] Mertz HR. Overview of functional gastrointestinal disorders: dysfunction of the brain-gut axis. Gastroenterol Clin North Am. (2003) 32:463–76. 10.1016/s0889-8553(03)00019-0 12858602

[12] Mayer EA Gebhart FG. Basic and clinical aspects of visceral hyperalgesia. Gastroenterology. (1994) 107:271–93. 10.1016/0016-5085(94)90086-8 8020671

[13] Camilleri M Testing the sensitivity hypothesis in practice: tools and methods, assumptions and pitfalls. Gut. (2002) 51(Suppl. 1):i34–40. 10.1136/gut.51.suppl_1.i34 12077062 PMC1867712

[14] Barbara G Cremon C De Giorgio R Dothel G Zecchi L Bellacosa L Mechanisms underlying visceral hypersensitivity in irritable bowel syndrome. Curr Gastroenterol Rep. (2011) 13:308–15. 10.1007/s11894-011-0195-7 21537962

[15] Delvaux M. Role of visceral sensitivity in the pathophysiology of irritable bowel syndrome. Gut. (2002) 51(Suppl. 1):i67–71. 10.1136/gut.51.suppl_1.i67 12077070 PMC1867713

[16] Mertz H Review article: visceral hypersensitivity. Aliment Pharmacol Ther. (2003) 17:623–33. 10.1046/j.1365-2036.2003.01447.x 12641510

[17] Ridding MC Ziemann U. Determinants of the induction of cortical plasticity by non-invasive brain stimulation in healthy subjects. J Physiol. (2010) 588:2291–304. 10.1113/jphysiol.2010.190314 20478978 PMC2915507

[18] Hobday DI Hobson AR Sarkar S Furlong PL Thompson DG Aziz Q. Cortical processing of human gut sensation: an evoked potential study. Am J Physiol Gastrointest Liver Physiol. (2002) 283:G335–9. 10.1152/ajpgi.00230.2001 12121880

[19] Otto SD Clewing JM Gröne J Buhr HJ Kroesen AJ. Repeatability of anorectal manometry in healthy volunteers and patients. J Surg Res. (2013) 185:e85–92. 10.1016/j.jss.2013.06.008 23968807

[20] Chaliha C Sultan AH Emmanuel AV Normal ranges for anorectal manometry and sensation in women of reproductive age. Colorectal Dis. (2007) 9:839–44. 10.1111/j.1463-1318.2007.01212.x 17509053

[21] Ooijevaar RE Felt-Bersma RJF Han-Geurts IJ van Reijn D Vollebregt PF Molenaar CBH. Botox treatment in patients with chronic functional anorectal pain: experiences of a tertiary referral proctology clinic. Tech Coloproctol. (2019) 23:239–44. 10.1007/s10151-019-01945-8 30778784 PMC6511340

[22] Rongqing G Yafei W Zhimin W Feng L Yuantao L Xinhua C. Treatment outcome of acute sacral nerve stimulation in functional anorectal pain. Pain Pract. (2019) 19:390–6. 10.1111/papr.12751 30472789

[23] Takano S Arakawa H. Bilateral posterior tibial nerve stimulation for functional anorectal pain–short term outcome. Int J Colorectal Dis. (2016) 31:1053–4. 10.1007/s00384-015-2380-x 26362818

[24] Bharucha AE Trabuco E Functional and chronic anorectal and pelvic disorders. Gastroenterol Clin North Am. (2008) 37:685–96. 10.1016/j.gtc.2008.06.002 18794003 PMC2676775

[25] Carrington EV Scott SM Bharucha A Mion F Remes-Troche JM Malcolm A. Expert consensus document: advances in the evaluation of anorectal function. Nat Rev Gastroenterol Hepatol. (2018) 15:309–23. 10.1038/nrgastro.2018.27 29636555 PMC6028941

[26] Eckardt VF Dodt O Kanzler G Bernhard G. Anorectal function and morphology in patients with sporadic proctalgia fugax. Dis Colon Rectum. (1996) 39:755–62. 10.1007/bf02054440 8674367

[27] Egorov V Lucente V van Raalte H Sarvazyan N. Biomechanical mapping of the female pelvic floor: changes with age, parity and weight. Pelviperineology. (2019) 38:3–11. 31341548 PMC6656381

[28] van Delft K Sultan AH Thakar R Schwertner-Tiepelmann N Kluivers K The relationship between postpartum levator ani muscle avulsion and signs and symptoms of pelvic floor dysfunction. BJOG. (2014) 121:1164–72. 10.1111/1471-0528.12666 24548759

[29] Harris ML Hobson AR Hamdy S Thompson DG Akkermans LM Aziz Q. Neurophysiological evaluation of healthy human anorectal sensation. Am J Physiol Gastrointest Liver Physiol. (2006) 291:G950–8. 10.1152/ajpgi.00010.2006 16690905

[30] Chan YK Herkes GK Badcock C Evans PR Bennett E Kellow JE. Alterations in cerebral potentials evoked by rectal distension in irritable bowel syndrome. Am J Gastroenterol. (2001) 96:2413–7. 10.1111/j.1572-0241.2001.04088.x 11513183

[31] Burgell RE Lelic D Carrington EV Lunniss PJ Olesen SS Surguy S. Assessment of rectal afferent neuronal function and brain activity in patients with constipation and rectal hyposensitivity. Neurogastroenterol Motil. (2013) 25:260–7. 10.1111/nmo.12047 23240734

[32] Giani I Novelli E Martina S Clerico G Luc AR Trompetto M. The effect of sacral nerve modulation on cerebral evoked potential latency in fecal incontinence and constipation. Ann Surg. (2011) 254:90–6. 10.1097/SLA.0b013e3182196ff4 21494120

[33] Knowles CH. Human studies of anorectal sensory function. Ir J Med Sci. (2018) 187:1143–47. 10.1007/s11845-018-1847-5 29926337 PMC6209020

[34] Törnblom H Van Oudenhove L Tack J Simrén M. Interaction between preprandial and postprandial rectal sensory and motor abnormalities in IBS. Gut. (2014) 63:1441–9. 10.1136/gutjnl-2013-305853 24142965

[35] Burgell RE Scott SM. Rectal hyposensitivity. J Neurogastroenterol Motil. (2012) 18:373–84. 10.5056/jnm.2012.18.4.373 23105997 PMC3479250

[36] Sangwan YP Solla JA Internal anal sphincter: advances and insights. Dis Colon Rectum. (1998) 41:1297–311. 10.1007/bf02258232 9788395

[37] Meunier P Mollard P. Control of the internal anal sphincter (manometric study with human subjects). Pflugers Arch. (1977) 370:233–9. 10.1007/bf00585532 563054

[38] Carlstedt A Nordgren S Fasth S Appelgren L Hultén L Sympathetic nervous influence on the internal anal sphincter and rectum in man. Int J Colorectal Dis. (1988) 3:90–95. 10.1007/bf01645312 3411187

[39] Beuret-Blanquart F Weber J Gouverneur JP Demangeon S Denis P. Colonic transit time and anorectal manometric anomalies in 19 patients with complete transection of the spinal cord. J Auton Nerv Syst. (1990) 30:199–207. 10.1016/0165-1838(90)90251-d 2229888

